# Comparison study on effectiveness of pentoxifyllin with LD to prevent recurrent endometriosis

**Published:** 2012-05

**Authors:** Ataollah Ghahiri, Aida Najafian, Mojdeh Ghasemi, Alireza Najafian

**Affiliations:** 1 *Department of Obstetrics and Gynecology, Isfahan University of Medical Sciences, Isfahan, Iran.*; 2 *Research Office of Shahid Beheshti Hospital, Isfahan University of Medical Sciences, Isfahan, Iran.*; 3 *Research assistant, Isfahan Endocrine and Metabolism Research Center (IEMRC), Isfahan, Iran.*

**Keywords:** *Endometriosis*, *Penthoxyfillin*, *Combined contraceptive pill (low dose estrogen) (LD)*

## Abstract

**Background: **About 75% of the symptomatic patients who involved with endometriosis have pelvic pain and dysmenorrhea. Pentoxifyllin is one of the drugs that according to its mechanism could be effective for pain relief of endometriosis which has been used for endometriosis treatment recently.

**Objective:** We conducted a comparative study for detecting the effect of pentoxifylin (as an immonomodelator) in preventing recurrence endometriotic pain with pentoxifylin plus a combined contraceptive pill with low dose estrogen (LD) and also the LD pill alone.

**Materials and Methods:** This was a comparative clinical trial on 83 patients with the chief complaint (CC) of pain (dysmenorrheal /or pelvic pain) and with the end diagnosis of endometriosis, in an operative laparoscopy. Patients, dividing to 3 groups, were treated with pentoxifylin, pentoxifylin+LD and LD alone for 10 months. The severity of pain (dismenorhea and/or pelvic pain) was detected by visual analogue scale (VAS) before and after the treatment. The severity of endometriosis in the patients was: I in class I and II in class II and III in class III. The groups were matched for the pain. The number of the patients in group 1, 2 and 3 were 28, 28 and 27 respectively.

**Results:** The pain was reduced in the groups of pentoxifylin+LD (p<0.001) and LD alone (p=0.00). The pain relief was not significant in the group of pentoxifylin alone (p=0.136). After treatment, the severity of pain was not significantly different between the LD group and the LD+penthoxyfillin group, but there was difference between these two groups and the group of penthoxyfillin alone.

**Conclusion:** This study showed that penthoxyfillin actually could not have any effect on the pain relief of endometriosis. It also made it clear that penthoxyfillin could not increase the efficacy of LD when used with this medication.

## Introduction

Endometriosis is the presence of the endometrial tissue (stroma and the glands) somewhere outside of the endometrial cavity. It can have debilitating symptoms like dysmenorrheal and pelvic pain or can be symptom less and discovered accidentally at laparoscopy (1). About 75% of the symptomatic patients has pelvic pain and dysmenorhea (2). 

The management of endometriosis is dependent to the managing physician and is chosen by the different factors like: the severity of symptoms, localization of the lesions, desire for pregnancy, age, and manifestation of the medications, surgical complications and finally the expense. Although most of the studies confirm the positive effect of the OCP in reducing the prevalence of endometriosis, but some studies express that it has no effect or even may cause increasing the rate of the disorder (3-5). 

In a controlled clinical trial, the effect of the OCP was compared with GNRH agonist. Both had good effects on recovering the pain of endometriosis, while the GNRH agonist showed better effects on treating the dyspareunia (6-15). One of the hypotheses of the cause of endometriosis is the immunologic change (16). With regard to this mechanism, using the immonomedulators can be a new method of managing endometriosis. This kind of medications can affect the immunity system at the site of peritonium, and one of the most popular drugs of this sort is pentoxifyllin which have been used in many studies (17). 

Pentoxifyllin is a methylgesantins suppressor of phosphodiesterase and possibly acts as an anti-inflammatory for the treatment of endometriosis. In addition to dysmenorrhea and pelvic pain, we can think of infertility as an important complication of endometriosis. Different studies have different results about the effect of this medication on infertility. So our study was conducted to compare the rule of pentoxifilin with OCP (LD) as a proved drug for the prevention of recurrence of endometriosis.

## Materials and methods

This was an open-label comparative clinical trial on 83 patients with the chief complaint of dysmenorrhea and or pelvic pain that underwent laparoscopic treatment of endometriosis in a private clinic and its aim was to compare the recurrence rate of the endometrioid symptoms. After obtaining informed consent, patients were randomly allocated to three groups based on the patients file number. Groups 1 and 2 were consisted of 28 patients, and group 3 had 27 patients. 

**Figure 1:**
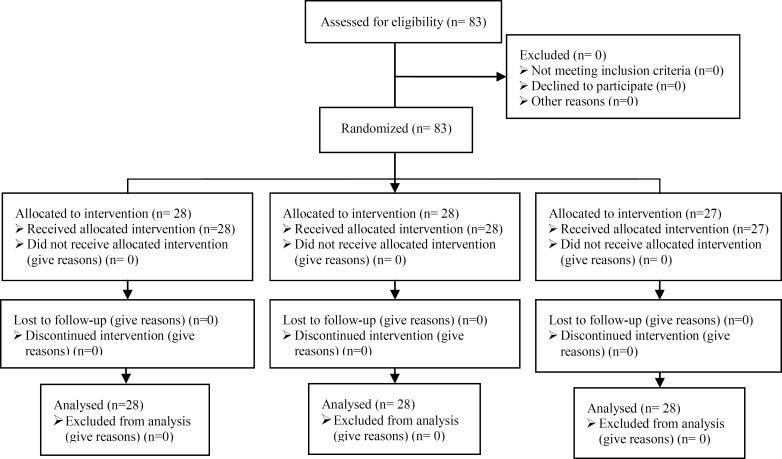
Consort flow chart of RCT

The patients were divided into three groups selecting by the simple randomization and after signing the consent form of each group. The number of the patients in groups 1, 2 and 3 were 28, 28 and 27 respectively. Inclusion criteria were mainly the diagnosis of pelvic endometriosis with the clinical symptoms and a full treatment procedure of cystectomy of possible endometrioma was done and/or fulguration of the endometriotic lesions by a bipolar coagulator (Figure 1). 

The exclusion criteria were refusing the patient of entry to the protocol or the presence of the specific drug manifestation. Each protocol was described clearly for each patient and finally the three groups were constructed as below:

- Group 1: 800 mgs of pentoxifyllin in two divided doses (capsules of 400 mgs) daily.

- Group 2: Pentoxifyllin capsules with the same dose plus OCP (LD) one tablet per night, from the 3rd day of the menstrual cycle.

- Group 3: OCP (LD) one tablet per night. Each treatment duration was about 6 months and no patient knew anything about the other group’s regimen.

**Statistical analysis**

We used the visual analog scale (VAS) method for detecting the severity of the possible pain existence before and after the treatment period (the recurrence). We also used the one way ANOVA and "post Hoc" statistical analysis test for comparing the results of the study and also for comparing the demographic data of the patient’s. P<0.05 was considered "significant" and SPSS 11.5 was used for the data analysis.

## Results

The mean age of the patients in group one was 27.5±5.5 yr, in group two was 25.27±6.7 yr and in group three was 27.01±4.68 yr. The average mean age was 26.59±4.70 yr with no significant difference between 3 groups (p=0.73). Pain severity comparing before starting the treatment regimen in 3 groups showed no statistically difference (p=0.47). But this comparison before and after the treatment in group one showed a significant difference; 7.67±1.41 vs. 3.64±2.81 respectively (p<0.001) (Table I). 

This was also seen in group two; before (7.70±1.61) and after (3.67±2.70) (p<0.001). In group 3, there was not any significant difference between before and after the treatment; 7.44±1.55 vs. 7.00±1.96 (p=0.136). At the end of the treatment regimen, pain severity comparison shared no significant difference between group one and two, while having significant difference between group three, and one and two (p<0.001).

**Table I:** Comparison of mean pain score before and after treatment in 3 groups

**Group **	**Pain score mean ** **before treatment**	**Pain score mean ** **after treatment**	**p-value**	**Number of patient in ** **each group**
LD	7.67+1.41	3.64+2.81	0.000	28
Combined	7.70+1.61	3.67+2.70	0.000	28
Pentoxyfillin	7.44+1.55	7.00+1.96	0.136	27

## Discussion

Pain is the most common symptom of endometriosis and about 75% of the symptomatic patients experience dysmenorrhea and pelvic pain. Medical treatment with different regimens has been suggested after the surgery (laparoscopy), to eradicate the possible remnant endometriotic tissue, and to reduce the recurrence of pain (17). Between the multiple regimens, OCPs are mostly used for treating endometriosis (7-10).

The results of our study showed that from these 3 regimens, pentoxifyllin+OCP (LD) and OCP (LD) alone were effective for pain release, and the pain severity scale before and after the treatment were statistically significant; but these two regimens were not significantly different for the pain release. 

Endometriosis related pain release by pentoxifyllin alone before and after the treatment was nadir and not statistically significant. So, by the above mentioned results, we can say that our study showed satisfactory effects of pain release of endometriosis by OCP (LD), but unsatisfactory effects by pentoxifyllin. And also, the combination of pentoxifyllin with OCP (LD), could not increase the positive results of the pill. 

Crosignani and his colleagues proved the positive immonumedulatory effects of pentoxifyllin for infertility caused by endometriosis, at their own study, but they also concluded that their results were just a beginning to this field and more studies were needed (18). Balash *et al* found no therapeutic effect for pentoxifyllin in treating the infertility induce endometriosis (19). Alborzi reported the effect of pentoxifyllin on the patients with endometriosis after a laparoscopy treatment in comparison to placebo. He found no therapeutic effect of pentoxifyllin on infertility and other symptoms of the endometriotic patients and its effect was equal to the placebo (20).

But a prospective randomized study on the patients with moderate to severe dysmenorrheal caused by endometriosis showed that the OCP could reduce the pain of these patients completely better than the placebo for 4 cycle treatment (9). Most of these studies during these years conclude that although the pantozifyllin is effective as an immonumodolator on animal studies, but no specific randomized clinical trial can prove the effect of pentoxifyllin on the symptoms of endometriosis on humans.

## Footnote

***This article extracted from residential thesis.***

***Registration ID in IRCT: IRCT201109087513N1***
